# A TLR4 agonist improves immune checkpoint blockade treatment by increasing the ratio of effector to regulatory cells within the tumor microenvironment

**DOI:** 10.1038/s41598-021-94837-7

**Published:** 2021-07-28

**Authors:** A. Farias, A. Soto, F. Puttur, C. J. Goldin, S. Sosa, C. Gil, F. A. Goldbaum, P. M. Berguer

**Affiliations:** 1 Fundación Instituto Leloir, IIBBA, Consejo Nacional de Investigaciones Científicas y Técnicas (CONICET), Buenos Aires, Argentina; 2 Inflammation, Repair and Development, National Heart and Lung Institute, Imperial College London, London, UK

**Keywords:** Cancer, Immunology

## Abstract

Brucella lumazine synthase (BLS) is a homodecameric protein that activates dendritic cells via toll like receptor 4, inducing the secretion of pro-inflammatory cytokines and chemokines. We have previously shown that BLS has a therapeutic effect in B16 melanoma-bearing mice only when administered at early stages of tumor growth. In this work, we study the mechanisms underlying the therapeutic effect of BLS, by analyzing the tumor microenvironment. Administration of BLS at early stages of tumor growth induces high levels of serum IFN-γ, as well as an increment of hematopoietic immune cells within the tumor. Moreover, BLS-treatment increases the ratio of effector to regulatory cells. However, all treated mice eventually succumb to the tumors. Therefore, we combined BLS administration with anti-PD-1 treatment. Combined treatment increases the outcome of both monotherapies. In conclusion, we show that the absence of the therapeutic effect at late stages of tumor growth correlates with low levels of serum IFN-γ and lower infiltration of immune cells in the tumor, both of which are essential to delay tumor growth. Furthermore, the combined treatment of BLS and PD-1 blockade shows that BLS could be exploited as an essential immunomodulator in combination therapy with an immune checkpoint blockade to treat skin cancer.

## Introduction

Cutaneous melanoma is a malignant tumor of melanocytic origin, with a high capacity to metastasize. Approximately 80% of all skin cancer-related deaths are attributed to melanoma although it comprises only 5% of all skin cancers^1^. In the past few years, immunotherapies have become the standard treatment regimens for patients with advanced melanoma^2^.

Immune checkpoint blockade (ICB) has emerged as a promising immunotherapy for cancer patients. This therapy enables tumor-reactive T cells to overcome regulatory mechanisms and mount an effective anti-tumoral response by removing inhibitory signals of T-cell activation. In steady state physiological conditions, these regulatory mechanisms are needed to avoid autoimmune responses and to control the magnitude of the immune response^3^. The most widely studied molecules involved in these processes are the anti-cytotoxic T-lymphocyte antigen-4 (CTLA-4), which dampens T cell activation by negative costimulation, and the programmed cell death protein 1 (PD-1), which maintains T-cell responses within a desired physiological range. Therefore, several antibodies against these proteins have been designed and FDA-approved for treatment against numerous types of tumors^3^. These antibodies are perceived to mediate anti-tumor activity by blocking CTLA-4 or PD-1/PD-L1 signaling pathways, releasing the suppression on effector cells^4^. Therefore, the efficacy of ICB relies on the infiltration of immune cells into the tumors^5^. However, to date, these treatments have only been shown to provide durable clinical benefit in a fraction of patients. As a consequence, combination regimen have recently emerged as a powerful immunotherapeutic strategy to benefit numerous cancer patients^4,6,7^. Combination therapies with Toll-Like Receptor (TLR) agonists and radiotherapy are of particular interest in this area of research. It is known that radiotherapy releases a large amount of tumor antigens that can be taken up by circulating dendritic cells (DC), inducing a tumor-specific immune response^8^. Moreover, TLR agonists induce activation of DC, promote Th1-type immune responses, antigen presentation and type I interferon production^9^. This potent immunomodulatory effect of TLR agonists, in combination with tumor-associated antigens can induce a specific anti-tumor response^10–12^. Several TLR agonists have been approved for their use in cancer therapy, such as Bacillus Calmette-Guérin (BCG) a TLR2/4 agonist, the TLR4 agonist monophosphoryl lipid A and the TLR7 agonist imiquimod. Furthermore, other TLR agonists have been studied and are undergoing pre-clinical and clinical evaluation^13,14^. Combination strategies using TLR agonists and ICB have been recently proposed. The rationale behind this combination is that the increased priming of antigen-presenting cells can potentially overcome resistance to PD-1/PD-L1 blockade and therefore enhance T cell activation. Several combinations have been reported to be beneficial in pre-clinical models. Even though there are several clinical trials ongoing based on TLR agonists and ICB, more research is needed to better understand how ICB can be modulated by TLR agonists.

We have previously shown that Lumazine Synthase from Brucella abortus (BLS), a highly stable decameric protein^15,16^, activates DC via TLR4, inducing the upregulation of costimulatory molecules and the secretion of proinflammatory cytokines and several chemokines^17^. Immunization with one dose of BLS induces the recruitment of B lymphocytes, CD4^+^ and CD8^+^ T cells and DC to the draining lymph nodes, as well as the expression of IFN-γ in the same lymphatic tissue^18^. Furthermore, we have previously reported that BLS has a therapeutic effect in B16 melanoma bearing mice when administered at early stages of tumor growth^19^. Here we demonstrate that this effect is, predominantly, due to the increment of tumor infiltrating leucocytes (TIL, identified as CD45^+^ cells). In particular, we show that treatment with BLS at day 2 increases the infiltration of effector T cells and decreases the proportion of regulatory T cells (Treg) and myeloid-derived suppressor cells (MDSC). Moreover, we show that combination of BLS with anti-PD-1 treatment boosts the antitumoral effect of BLS.

## Results

### BLS expands tumor infiltrating leucocytes when administered at early stages of tumor growth

**Figure 1:**
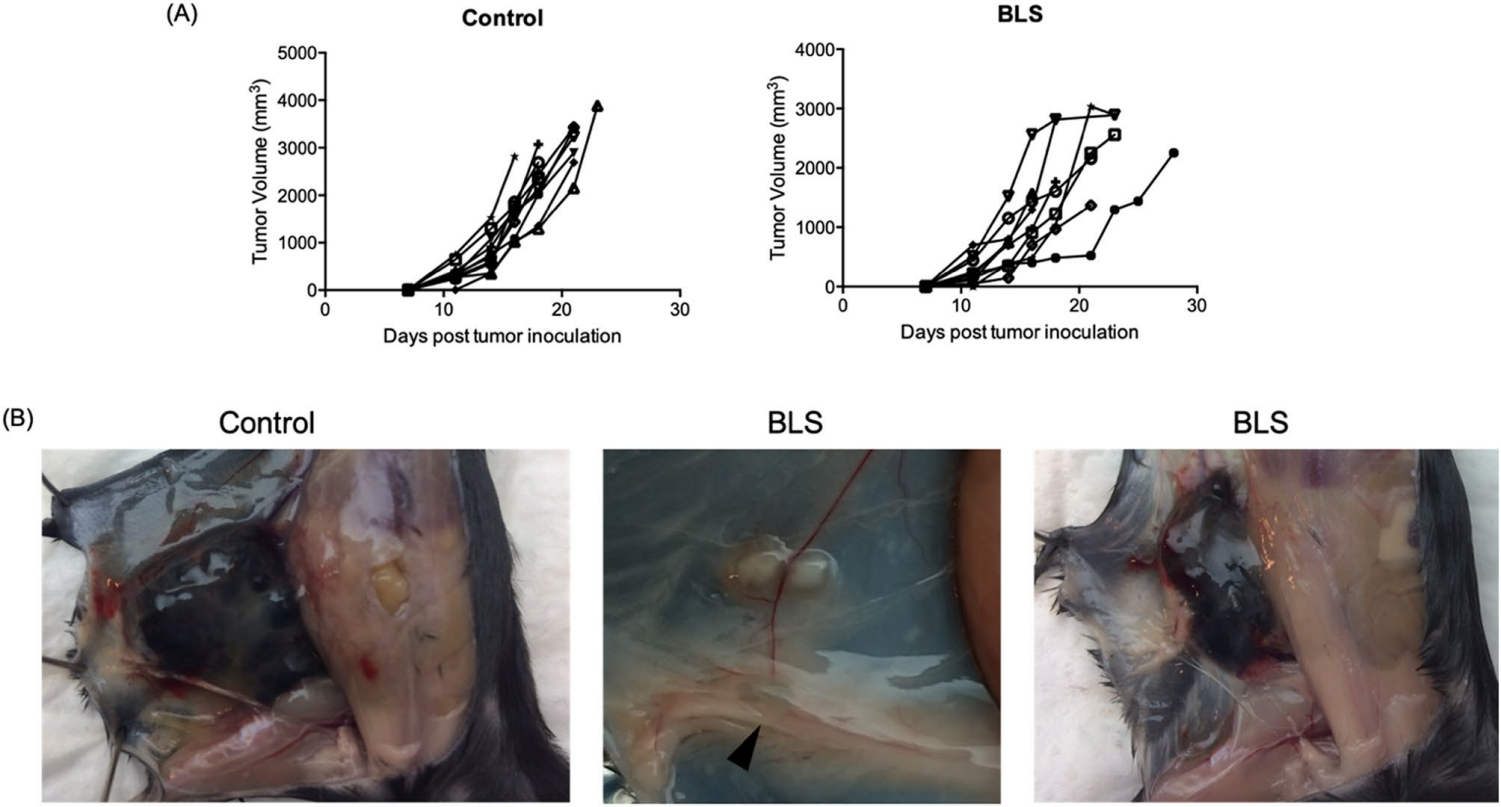
BLS treatment delays tumor growth. C57BL/6J mice were inoculated with 1.25 × 10^5^ B16 cells subcutaneously in the right flank. At day 2, 200 μg of BLS or PBS (control) were subcutaneously administered in the base of the tail. Tumor growth (**A**) was followed every 2–3 days and tumor volume was estimated as 0.5 × (length × width^2^). Data pooled from two independent experiments (n = 4 or 5 mice per group per experiment). Representative photos of a control tumor and a photo the smallest and biggest tumors from BLS treated mice at day 14 after tumor inoculation are shown (**B**). The arrow shows the tumor draining lymph node.

The B16 melanoma is a poorly immunogenic model, characterized with a low proportion of tumor infiltrating leucocytes composed mainly of MDSC, which block T cell infiltration and leads to immune escape^20^. It has been established for this model that a higher ratio of effector T cells (especially Cytotoxic T cells) to regulatory cells (including regulatory T cells and MDSC) within the tumor microenvironment is a pivotal prognostic marker for cancer^21^. We have previously shown that treatment with BLS at day 2 has a therapeutic effect in melanoma bearing mice (Fig. 1A and^19^). Nevertheless, BLS is not able to induce this therapeutic effect when treatment is carried out at day 10. To better understand the mechanisms underlying the effect triggered by BLS, we studied the composition of the tumor microenvironment. B16 cells were subcutaneously (s.c.) inoculated in C57BL/6J mice and at day 2 or 10, BLS was administered s.c.. At day 14, when all mice had palpable tumors (Fig. 1B), we evaluated the proportion of CD45^+^ cells (referred as TIL) in the TME by flow cytometry (Fig. S1).

**Figure 2:**
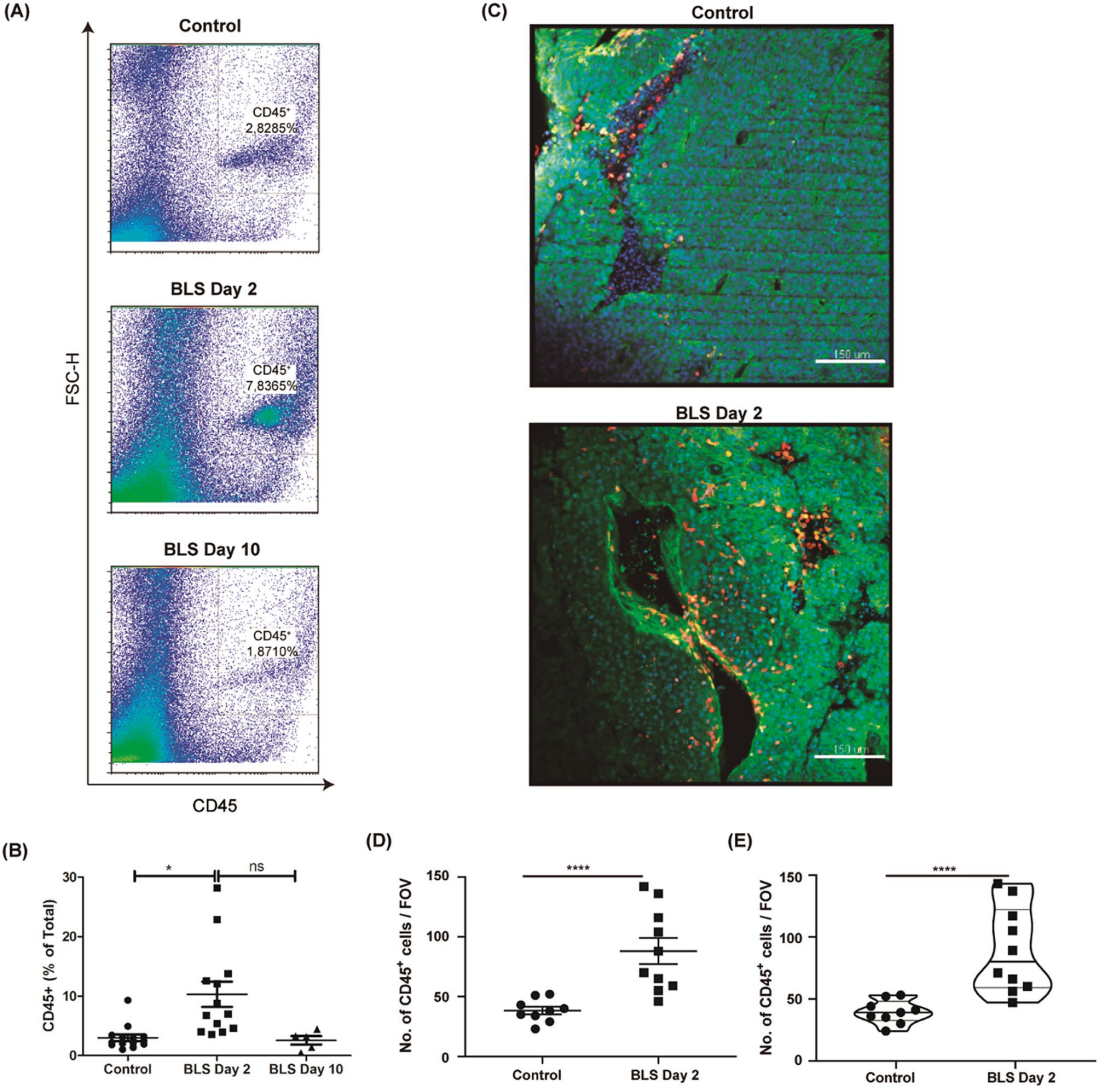
BLS administration impacts in the tumor microenvironment composition. C57BL/6J mice were inoculated with 1.25 × 10^5^ B16 cells subcutaneously in the right flank. At day 2 or 10, 200 μg of BLS or PBS (control) were subcutaneously administered in the base of the tail. At day 14 the abundance of CD45^+^ cells within the tumor was analyzed by flow cytometry as depicted in representative FACS plots (**A**) and quantified as % of Total live cells in (**B**). For (**C**–**E**), fixed precision cut tumor slices of 200 µm thickness were obtained and stained for phalloidin (Green, the tumor structure and blood vessels), CD45 (Red), and static z- stack snapshots were captured by taking a 1024 µm × 1024 µm field of view (FOV), under a ×20 dry objective using an inverted confocal microscope (**C**). In (**D**) number of CD45^+^ cells were quantified in a dot plot and violin plot (**E**) by compared between BLS-day 2 treatment and control (pooled data where each dot represents an individual tumor slice and 3 slices per individual mouse tumor). Data are representative from 2 individual or four independent experiments have been pooled (n = 3 mice per group per experiment). *p < 0.05.

The percentage of TIL in control tumors represented less than 5% of the tumor mass. Interestingly, tumors from mice treated with BLS at day 2 had an increased percentage of TIL (Fig. 2A,B). In combination with the decreased volume, the increased frequency of TIL in the tumors from BLS treated mice suggest an increased local immune response. We next set out to evaluate the anatomical distribution of CD45^+^ cells within the 3D tumor microenvironment. The increase in proportion of CD45^+^ cells after BLS treatment at day 2 could be visualized by imaging fluorescently labelled CD45^+^ cells in precision cut tumor slices (Fig. 2C) and quantified (Fig. 2D). The increase in CD45^+^ cells was consistent across individual tumor slices obtained from different mice (Fig. 2E). This increment of TIL was not detected in mice treated at day 10. These results show that treatment with BLS only at early stages of tumor growth impacts the TME composition, increasing the percentage of leucocytes. Overall, our results show that BLS treatment at day 2 significantly increases the percentage of CD45^+^ leukocytes compared to control mice.

### Administration of BLS in tumor-bearing mice induces a transient secretion of serum IFN-γ

**Figure 3:**
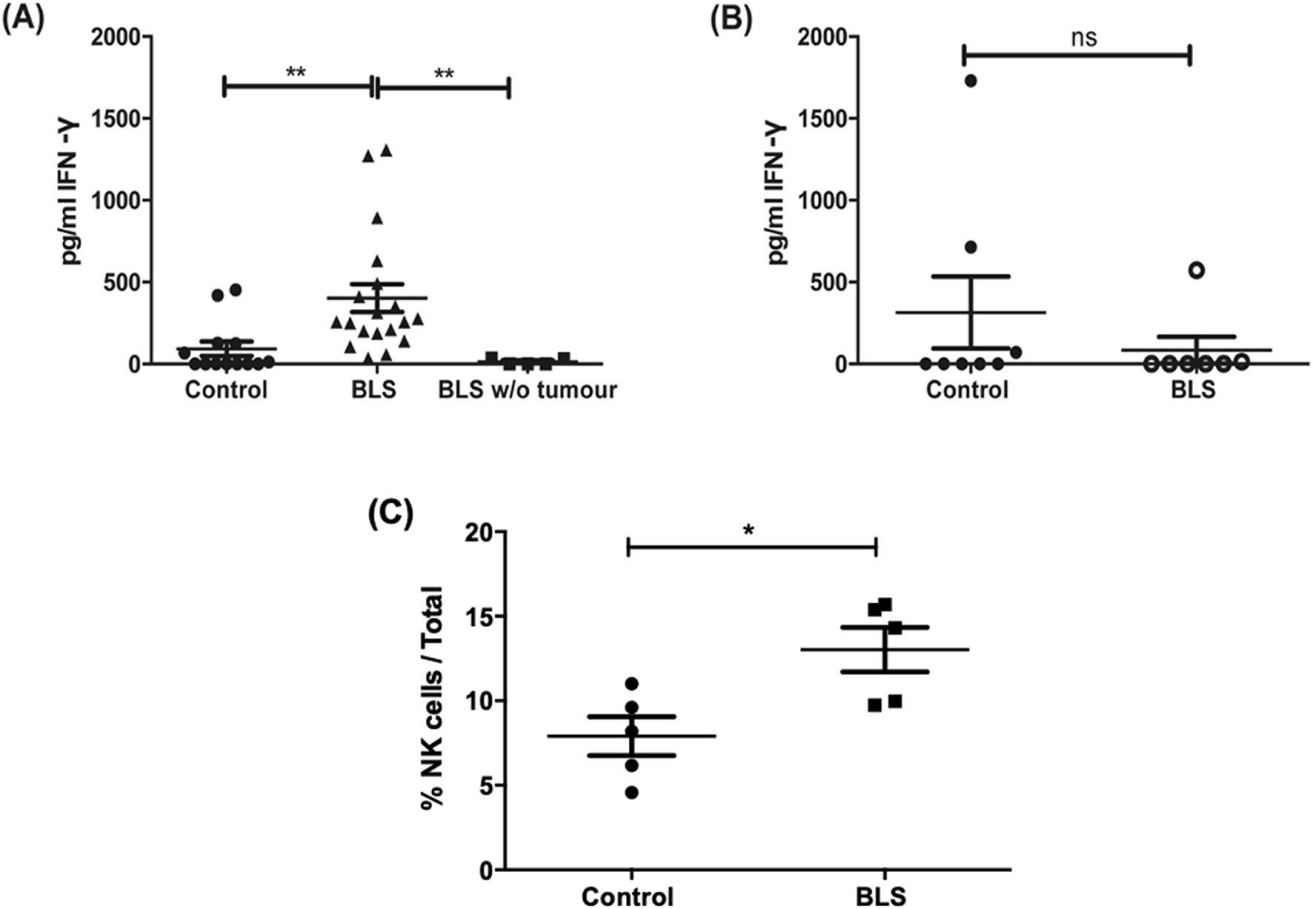
Early administration of BLS induces the secretion of systemic IFN-γ as well as an increase in the proportion of circulating NK cells. C57BL/6J mice were inoculated with 1.25 × 10^5^ B16 cells subcutaneously in the right flank. At day 2 (**A**) or 10 (**B**) 200 μg of BLS or PBS (control) were subcutaneously administered in the base of the tail. After 3 h blood was obtained, Levels of IFN-γ in serum were analyzed by ELISA and abundance of NK cells was assessed by flow cytometry (**C**). Data from two to four independent experiments have been pooled ( n= 4 or 5 mice per group per experiment), **p < 0.005.

An effective anti-tumor response is mediated by both the innate and adaptive immune response. Natural killer cells (NK cells) play an important role during cancer immunity, as they can detect and kill malignant cells. Moreover, NK cells can orchestrate the adaptive immune response by secreting IFN-γ. Since IFN-γ plays a crucial function in generating antitumoral responses, not only by inducing T cell attractant chemokines, but also polarizing the immune response to a Th1 profile^22^, we evaluated the expression of this protein following treatment with BLS. To address this aim, serum samples were collected 3 h after BLS administration and levels of IFN-γ were determined by ELISA. Our results show that treatment with BLS at day 2 (Fig. 3A) but not at day 10 (Fig. 3B) induces the expression of IFN-γ. In contrast, BLS fails to induce the secretion of IFN-γ in the serum of healthy mice. The levels of serum IFN-γ were not detectable in any group 24 h after treatment (Data not shown). Moreover, in vitro stimulation of B16 cells with BLS did not induce detectable levels of IFN-γ (Data not shown). We then analysed the proportion of circulating NK cells in mice inoculated with tumor cells and treated with BLS or PBS after 2 days (Fig. S2). In concordance with the levels of serum IFN- γ, an increase in the percentage of NK cells was detected in BLS-treated mice (Fig. 3C). Collectively, our results suggest that administration of BLS at early stages of tumor growth, induces an early expression of serum IFN-γ, most likely by NK cells, which may facilitate the antitumor response.

### BLS reduces the proportion of regulatory T and myeloid cells within B16 melanoma tumors

**Figure 4:**
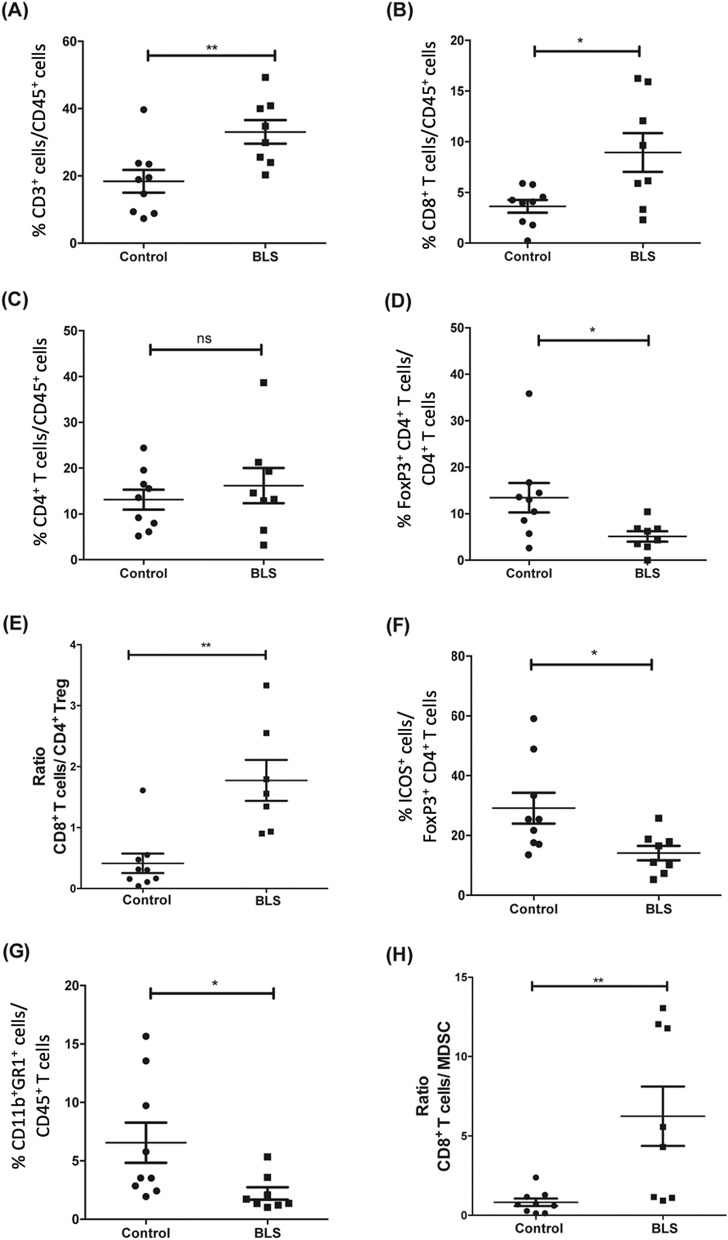
BLS administration impacts in the tumor microenvironment composition. C57BL/6J mice were inoculated with 1.25 × 10^5^ B16 cells subcutaneously in the right flank. At day 2, 200 μg of BLS or PBS (control) were subcutaneously administered in the base of the tail. At day 14 the percentage of CD3^+^ (**A**), CD8^+^ (**B**), CD4^+^ (**C**), Treg (**D**) within the CD45^+^ population present in the tumor were quantified by flow cytometry. Ratio of CD8^+^ T cells and Treg was calculated (**E**) and ICOS expression was assessed on Treg (**F**). Proportion of MDSC within CD45^+^ cells (**G**) and the abundance compared to CD8^+^ T cells was calculated for each mouse (**H**). Data from two independent experiments have been pooled ( n = 4 or 5 mice per group per experiment), *p < 0.05, **p < 0.01.

The B16 melanoma is a poorly immunogenic tumor, characterized by low proportion of TIL^23^. Furthermore, it has been described that the lack of CXCR3 expression results in increased tumor growth and even lower levels of infiltrating T cells^24^. Interestingly, the ligands for CXCR3 are the CXCL9, CXCL10 and CXCL11 chemokines, proteins known as IFN-dependent proteins. In this study we demonstrate that treatment with BLS at early stages of tumor growth initially induces the production of serum IFN-γ and modifies the TME by increasing the percentage of TIL. In order to further characterize the changes triggered by BLS in the TME, we identified the immune cells present within the tumors. Melanoma cells were inoculated, and at day 2 mice were treated with BLS or PBS. At day 14, we analyzed them by flow cytometry (Fig. S3). BLS treatment strongly increased the infiltration of CD3^+^ T cells in the tumor (Fig. 4A). Among the tumor infiltrating T cells, CD8^+^ T cells were the primary T cell subset (Fig. 4B). In contrast, the percentage of CD4^+^ T cells from the tumors of BLS treated mice remained comparable to the percentage of CD4^+^ T cells observed in control tumors (Fig. 4C).

Since Treg cells are often detected in inflamed tumors harboring large numbers of CD4^+^ T helpers and CTL, we further evaluated the effects of BLS administration on Treg in the TME. In mice, expression of FoxP3 defines a T reg cell population. Therefore, we used the Foxp3^+^ CD4^+^ gating strategy, allowing us to also evaluate levels of expression of the immunosuppressive molecule Inducible T-Cell Costimulator (ICOS)^25^. Interestingly, we found that the fraction of Treg among CD4^+^ T cells decreases (Fig. 4D) after treatment with BLS, elevating the proportion of CD8^+^ T cell to Treg (Fig. 4E). Furthermore, the levels of ICOS expression in this population were lower compared to the control group (Fig. 4F).

Additionally, we assessed the effect of BLS therapy on MDSC using the traditional CD11b^+^GR1^+^ gating^21^. We show that BLS decreases the infiltration of MDSC (Fig. 4G), increasing the ratio of CD8^+^ T cells to MDSC (Fig. 4H).

Taken together, these results show that treatment with BLS greatly impacts on the TME, inducing the recruitment of CD8^+^ T cells, and decreasing the proportion of suppressor cells.

### Elucidating the effect of BLS on the PD-1/PD-L1 pathway

**Figure 5:**
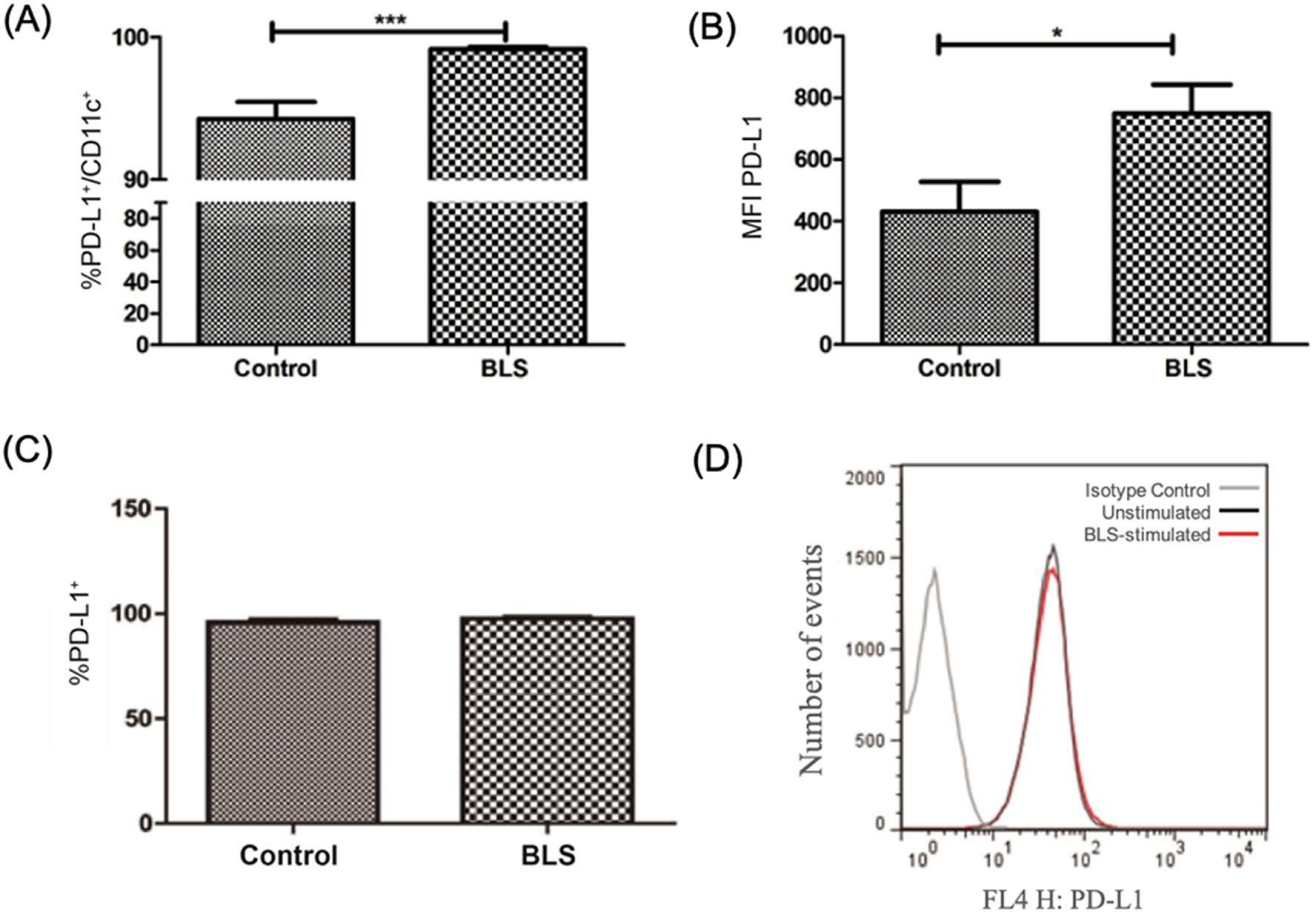
PD-L1 expression on BMDC and B16 cells upon BLS stimulation*.* BMDC from C57BL/6J (**A**, **B**) mice were generated and stimulated with BLS or non-stimulated (control) for 18 h. B16 cells (**C**, **D**) were cultured with BLS or non-stimulated (control) for 48 h. Level of expression of PD-L1 was evaluated by flow cytometry. Three different experiments were pooled (n = 2 replicates per experiment).

We have shown that treatment with BLS at early stages of tumor growth delays its progress and therefore increases survival. However, this treatment is not sufficient to eliminate tumor cells. Aside of the recruitment of different regulatory cells, other mechanisms, like the upregulation of immune checkpoints (such as PD-1, CTLA-4 and TIM-3), have been reported to help immune evasion^26^. We next investigated the PD-1/PD-L1 pathway, since inflammatory stimuli induce PD-L1 expression, not only in antigen presenting cells but also in many different type of tumor cells^26^. In particular, it has been suggested that the anti-tumoral effect of poly I:C is limited by the upregulation of PD-L1 on DC, due to the impact on the CD8^+^ T cell priming and activation^27^. Consequently, we evaluated if BLS was able to alter PD-L1 expression in activated DC. To that end, in vitro differentiated BMDC from wild type mice were generated using mGM-CSF and stimulated with 90 µg of BLS for 18 h, and expression of PD-L1 was studied by flow cytometry. An up-regulation of PD-L1 expression was observed upon activation with BLS (Fig. 5A,B) in a TLR4 dependent manner (data not shown). Hence, we show that in vitro, BLS induces an upregulation of PD-L1 upon activation of DC. Moreover, we studied the expression of PD-L1 in melanoma cells. The cell line used in this study express high levels of the PD-L1 ligand when cultured in vitro, and BLS treatment does not affect the level of PD-L1 expression (Fig. 5C,D). Taken together, these results suggest that combination of BLS and the blockade of the PD-1/PD-L1 pathway could increase the therapeutic effect of BLS in tumor-bearing mice.

### Combined BLS and anti-PD-1 therapy increases the antitumor effect

**Figure 6:**
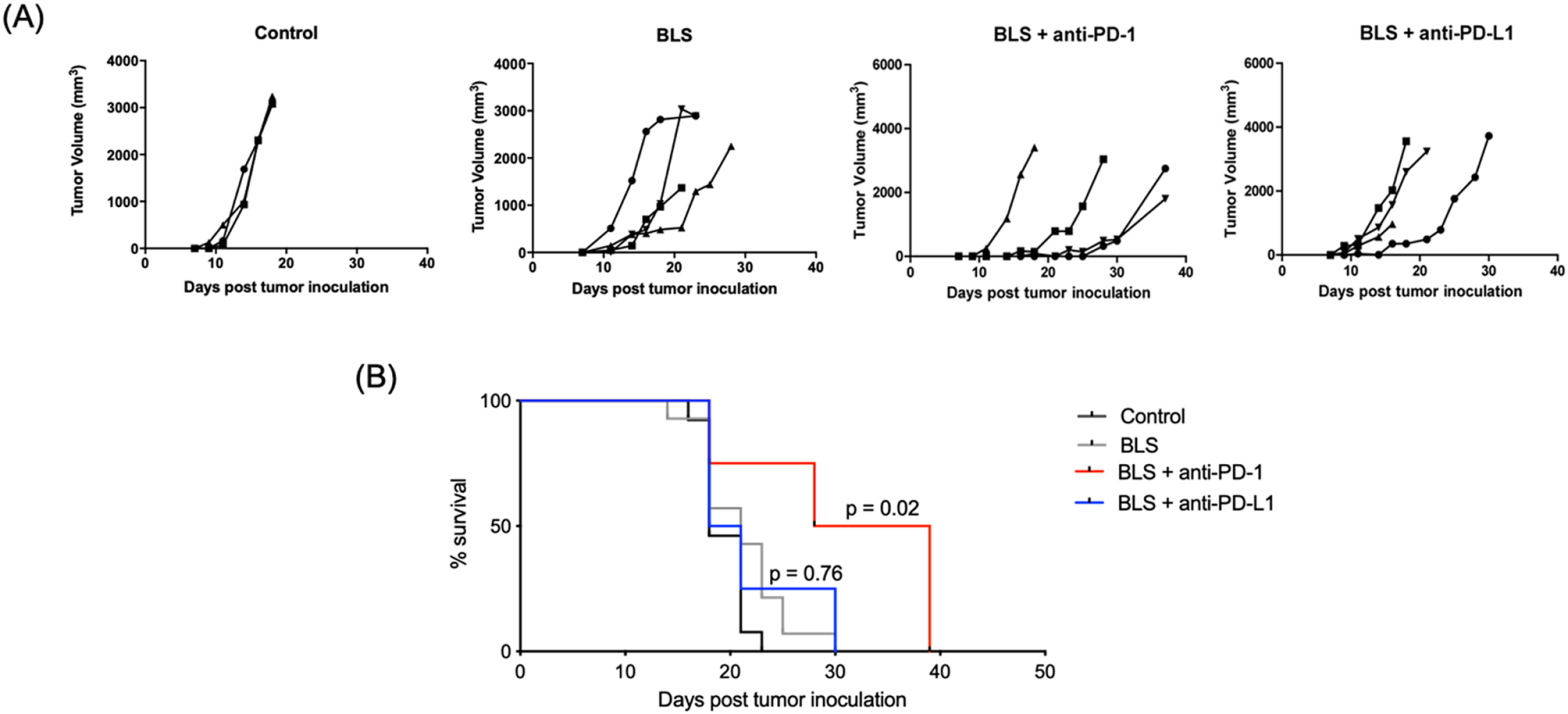
Combination treatment of BLS and PD-1/PD-L1 pathway blockade improves the outcome of BLS monotherapy. B16 cells were inoculated in C57BL/6J mice. At day 2, 200 μg of BLS were administered s.c., and at days 5, 8 and 11 200 µg of anti-PD-1 or anti-PD-L1 were i.p. inoculated. Tumor growth (**A**) and mice survival (**B**) were followed every 2–3 days and tumor volume was estimated as 0.5 × (length × width^2^). Results from one representative experiment (n = 4) are shown.

**Figure 7:**
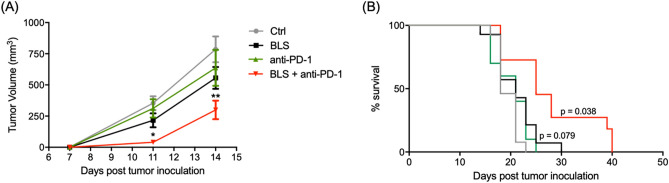
BLS treatment overcomes resistance to anti-PD-1 blockade. B16 cells were inoculated in C57BL/6J mice. At day 2, 200 μg of BLS or PBS (Ctrl and anti-PD-1) were administered s.c., and at days 5, 8 and 11, 200 µg of anti-PD-1 or PBS were i.p. inoculated. Tumor growth (**A**) and mice survival (**B**) were followed every 2–3 days and tumor volume was estimated as 0.5 × (length × width^2^). Results from two independent experiments were pooled (n = 7 mice per group per experiment). *p < 0.05; **p < 0.01.

We have shown that treatment with BLS at early stages of tumor growth induces the rapid secretion of systemic IFN-γ and a significant increment of TIL with an augmented ratio of effector to regulatory cells. IFN-γ-regulated genes have been associated with response to PD-1 blockade^24^ and a hot T cell-inflamed TME, as the one induced by BLS, correlates with responsiveness to ICB^28^. Moreover, the tumor cell line used in this work has a high expression of PD-L1 and activation of BMDC with BLS increases the expression of the ligand, suggesting that the PD-1/PD-L1 pathway can limit the therapeutic outcome. Thus, we hypothesized that combination with ICB would improve the therapeutic effect of BLS. To validate our hypothesis we combined BLS with anti-PD-1 or anti-PD-L1 treatment. Mice were inoculated with B16 cells, 2 days later BLS was administered s.c., and at days 5, 8 and 11 anti-PD-1 or anti-PD-L1 antibodies were intraperitoneally (i.p.) inoculated. Tumor growth was followed every 2–3 days, and tumor volume was estimated. Remarkably, combination with anti-PD-1, and not anti-PD-L1, delays tumor appearance and growth (Fig. 6A), significantly increasing mice survival compared to treatment only with BLS (Fig. 6B). Given these results, we next compared the efficacy of the combined treatment compared to anti-PD-1 blockade. The monotherapy with anti-PD-1 in this experimental set up does not influence tumor growth nor survival compared to the untreated group. Strikingly, combination of BLS and anti-PD-1 has a synergic effect, overcoming resistance to immune checkpoint blockade (Fig. 7).

In conclusion, our data shows that treatment with BLS modifies the tumor microenvironment by increasing the proportion of CD8^+^ T cells and therefore allowing a local anti-tumor response. Additionally, we demonstrated that the anti-tumoral mechanisms triggered by the immunomodulatory effects of BLS have a synergistic effect when combined with PD-1 blockade.

## Discussion

From the inception of cancer immunotherapy, amplifying anti-tumor responses has been the strategic choice of treatment^29^. However, recent evidence suggests that durable anti-tumor immune responses with low adverse side-effects may serve as a superior alternative towards improving treatment for cancers like metastatic melanoma^30^. The fact that the immune system is able to infiltrate and survey the tumor microenvironment has led to numerous studies that have improved our understanding on the biology of tumor immune regulatory pathways and development of novel therapies. These have included either overcoming inhibitory pathways, for example: checkpoint blockade such as anti-CTLA-4 and anti-PD-1, or stimulating immune cell activation pathways, such as co-stimulation with anti-GITR and anti-OX40^31^. In this respect, mouse models of melanoma have proved invaluable and contributed to a major part of the success of immunotherapy. Studies employing mouse models of melanoma have shown that targeting certain innate immune signaling pathways, in particular, TLR, retinoic acid-inducible gene-I-like receptors (RLR), and stimulator of interferon genes (STING) signaling pathways may prove critical towards aiding in tumor regression either by direct induction of tumor cell apoptosis^32^ or by reducing tumor cell proliferation^33^. In this respect, our previous work has shown that BLS administered to B16 tumor-bearing mice at the initial stages of tumor growth has a therapeutic effect. We have also shown that the lack of therapeutic effect when BLS is administered at later stages of tumor growth correlates with the decreased expression of TLR4 in the tumor cell surface. In consequence, we propose that the therapeutic effect triggered by BLS is due to its direct signaling on tumor cells via TLR4^19^. In this report we provide further evidence in support of our hypothesis. Firstly, we show that treatment with BLS at day 2 induces the recruitment of hematopoietic immune cells into the tumor. In contrast, inoculation of BLS at day 10 fails to increase the proportion of CD45^+^ cells in the TME. Moreover, a rapid release of IFN-γ was detected 3 h after treatment with BLS at day 2, with cytokine levels persisting at baseline levels when administered in mice with palpable tumors or in naïve mice. The secretion of IFN-γ coincided with increased NK cells in circulation in BLS treated mice compared to the untreated group. Taken together, these results suggest that the effectiveness of the early treatment with BLS can be attributed to its ability to modify the tumor microenvironment and to induce expression of IFN-γ by NK cells, only in the presence of B16 cells. In order to better understand the mechanisms underlying the therapeutic effect of BLS, we characterized the tumor microenvironment induced by treatment with BLS at early stage of tumor growth. We found that administration of only one dose of BLS is sufficient for inducing the trafficking of CD8^+^ T cells within the tumor. Interestingly, this therapy also decreases the percentage of Treg present in the tumor, increasing the ratio of CD8^+^ T cells to Treg cells and consequently increasing the antitumor response. Additionally, we found that ICOS expression on Treg is downregulated after treatment with BLS, suggesting that the tumor resident Treg from treated mice may be less suppressive than infiltrating Treg in tumors from control mice. Aside of Treg, we observed a pronounced accumulation of myeloid-derived suppressor cells within the tumor, suggesting an alternative tumor escape mechanisms^34^. Thus, we investigated if BLS also impacts the MDSC population. Remarkably, administration of BLS decreases the accumulation of MDSC within the tumor, increasing the ratio of effector to regulatory cells. Collectively, our results suggest that the therapeutic outcome of BLS is due to its ability to increase the proportion of CD8^+^ T cells compared to both Treg and MDSC, leading to a proper local anti-tumor response. Even though our study was focused on the adaptive anti-tumor response, these cells represent a small proportion of CD45^+^ population. Within the immune cells infiltrating the tumor, macrophages, dendritic cells, neutrophils and NK cells also play a key role in the anti or pro-tumoral response^22^. Interestingly, IFN-γ induces macrophage polarity to a pro-inflammatory profile increasing the anti-tumoral response. Similar effects of IFN-γ have been reported on neutrophils, shaping their role in the anti-tumoral response. NK cells play a major role in cancer immunosurveillance, as they can rapidly detect and kill malignant cells as well as mediate the innate and adaptive anti-tumor response^35^. Moreover, NK-secreted IFN-γ increases dendritic cells stimulatory capacity as well as antigen uptake and presentation, leading to a specific antitumoral adaptive immune response. It has been reported that activated NK cells can interact with stromal cells, leading to changes in the tumor architecture^36^. Therefore, it is possible that an early activation of NK cells after BLS treatment has an impact on tumor seeding and growth by modulating tumor architecture and increasing infiltration of immune cells within the tumor, improving the local antitumoral response.

We have demonstrated that BLS at early stages of tumor growth delays tumor development^19^; however all treated mice eventually succumb to the tumors. A possible mechanism involved in tumor evasion is the upregulation of immune checkpoints, such as PD-1, PD-L1 or CTLA-4. Activation of dendritic cells via TLR agonists, including BLS, has been shown to upregulate surface PD-L1 impacting in the magnitude of the CD8^+^ T cell response^37,38^. Intriguingly, administration of BLS followed by PD-L1 blockade unaltered the overall benefit of BLS. The lack of increased therapeutic benefit when combining BLS with anti-PD-L1, could be attributed to the time point of antibody administration, as 72 h after BLS treatment can be ideal to boost T cell response, but may be late to improve priming. Although all B16 cells in vitro express high levels of PD-L1, in the TME only 50% of the non-hematopoietic cells in the TME express PD-L1. Intriguingly, treatment with BLS decreases the expression of the ligand at the specified time point (Fig. S4). These results show that administration of BLS impacts in the level of expression of PD-L1 in the CD45^-^ cells from the TME. Thus, it is possible that the downregulation of the ligand within the TME also explains the different outcome between PD-1 and PD-L1 blockade. In line with these results, it has been described, for the B16 melanoma model, that PD-L1 expression is increased on tumor cells by direct contact with bone marrow CD11b^+^ cells^39^. After treatment with BLS, the frequency of CD11b^+^ cells within the tumor decreases, supporting the downregulation of surface PD-L1 on the non-hematopoietic cells within the TME.

Nevertheless, combination of BLS with anti-PD-1 significantly delays tumor growth increasing survival of mice compared to the monotherapies. This work verifies the immunomodulatory effects of BLS in B16 melanoma bearing mice. In order to speculate on the possible mechanisms underlying the effect of the combined treatment, we need to focus on how BLS is likely sensitizing the tumor by inducing an inflamed or hot TME, as it is now clear that immune checkpoint blockade efficacy relies on the immune cell infiltration in the TME^40^. In this respect, we have shown that BLS has a direct effect on B16 cells, inducing activation of the tumor cells and impacting on tumor growth in vivo. Moreover, serum IFN-γ was detected only when BLS is administered early after tumor inoculation. Increased IFN-γ synchronized with increased TIL in the TME, suggesting that both, the expression of IFN-γ and the high frequency of CD45^+^ cells in the TME contributed to delaying tumor growth. These results, in combination of the decreased expression of TLR4 at late stages of tumor growth (10 days after tumor inoculation) reported previously^19^, indicates that BLS can interact with the tumor cells in vivo through TLR4, inducing an antitumoral response. Moreover, BLS induces the activation of dendritic cells and the expression of proinflammatory cytokines and chemokines through TLR4 signaling^17^. The mechanisms underlying the therapeutic effect of BLS remains unclear. Whether the mechanism involves the direct interaction of BLS with the tumor cells, the host innate immune cells or a combination of both remains to be addressed in future studies. However, we hypothesized that the synergistic effect observed is due to the hot TME, characterized by an increased frequency of CD8^+^ T cells and decreased presence of regulatory cells, leading to the increased susceptibility to anti-PD-1 blockade.

In conclusion, the results presented in this work show that BLS serves as an adjuvant in combination with ICB. This TLR4 agonist emerges as a novel immunomodulator not only due to the adjuvant properties but also due to its distinctive carrier properties^41–43^. As previously reported, BLS not only can induce an immune response against covalently attached peptides, but it also increases the stability of these antigens, making BLS-chimeras easy to design and produce. We have previously showed that treatment with a chimera of BLS does not improve the therapeutic benefits of BLS^19^. Evidence in preclinical mouse models suggest that vaccination using synthetic long peptides (SLP) containing neoantigens^44–46^ improves the efficiency of immunotherapy^47^. Together, these data suggest that attachment of SLP to BLS could present a potentially feasible strategy to improve the therapeutic effect of BLS chimeras. Thus, BLS arises as a platform of personalized anti-tumor vaccines which, in combination with ICB, could increase the efficacy of immunotherapies in melanoma. Further studies are needed to validate the antitumoral effect by the chimeras of BLS in pre-clinical animal models and develop strategies to combine them with ICB, to maximize the therapeutic effects of BLS shown in this work.

## Materials and methods

### Protein purification

Cloning, recombinant expression, and purification of BLS protein were performed as described previously^16^. Briefly, the BLS gene was cloned into the pET11a vector (Novagen, Madison, USA) and transformed and expressed in inclusion bodies in the BL21 (DE3) strain of *Escherichia coli*. The inclusion bodies were solubilized in 50 mM Tris, 5 mM EDTA, and 8 M urea (pH 8.0) overnight at room temperature with agitation. The solubilized material was refolded by dialysis against PBS containing 1 mM DTT for 72 h. This preparation was purified with a Q-Sepharose column (Amersham Biosciences, Little Chalfont, UK) in a fast performance liquid chromatography apparatus (Amersham Biosciences, Little Chalfont, UK) using a linear gradient of NaCl between 0 and 1 M in a 50 mM Tris (pH 8.5) buffer. The peak enriched with BLS was further purified on a Superdex-200 column with PBS, 1 mM DTT. The purity of the BLS preparation was determined using 15% (w/v) SDS-PAGE. BLS was concentrated (to 7 mg/ml), frozen in liquid N_2_, and stored at − 80 °C. The purified protein was detoxified by incubating 1 mg of BLS with 500 μl of polymyxin B-agarose (PMB-agarose, Sigma-Aldrich, Saint Louis, MO, USA) overnight twice at 4 °C, as previously described^43^. Limulus amebocyte lysate (LAL) test was performed in order to assure that BLS preparations were free of LPS. Determinations were carried out following manufacturer’s instructions (Associates of Cape Cod. Rev 002. Nov 2003. LAL Pyrotell Multitest vial instruction sheet). Pyrotell LAL for gel-clot assay, LAL reagent water, endotoxin standard, tips and tubes were purchased from Associates of Cape Cod (Woods Hole, MA, USA).

### Mice and cell culture

C57BL/6J mice were obtained from The Jackson Laboratory and bred in the animal facility at Leloir Institute. All mice were bred under specific pathogen-free conditions and were used at 8–10 week of age. B16-F1 melanoma (ATCC CRL-6323, Manassas, Virginia, United States), syngeneic from C57BL/6 mice was a kind gift from Dr José Mordoh´s lab and was cultured at 37 °C under 5% CO_2_ in endotoxin-free RPMI 1640 medium supplemented with 10% FBS (Gibco; Grand Island, NY, USA), penicillin and streptomycin, 1 mM pyruvate and 4 mM l-glutamine.

### Tumor inoculation and vaccination

Mice were inoculated in the flank subcutaneously at day 0 with 1.25 × 10^5^ B16 cells. Tumor growth was monitored every 2 or 3 days and diameters were measured using a caliper. The major longitudinal diameter (length) and the major transverse diameter (width) were determined, and tumor volume was approximated based on caliper measurements by the following formula: tumor volume = 0.5 × (length × width^2^). For combined treatments, at day 2 mice were s.c. injected with 200 μg of BLS and at days 5, 8 and 11, 200 μg of α-PD-1 or α-PD-L1 (clones RMP1-14 and 10F.9G2 respectively, Bioxcell, Lebanon, Pennsylvania, USA).

### Determination of cytokines

BLS was administered 2 or 10 days after tumor inoculation. Treatment was also performed in healthy mice. Level of IFN-γ was measured in serum 3 h after BLS injection, using ELISA (all OptEIA sets; BD Pharmingen), following the manufacturer’s instructions. The reaction was developed by adding 50 µl of a solution containing 2 g/l ortho-phenylenediamine and 0.03% H_2_O_2_ in 0.1 M citrate–phosphate buffer and was stopped with 50 µl of 4 N H_2_SO_4_. The final color was read at 492 nm in an ELISA reader (SLT Lab Instruments). The detection limit was 31.3 pg/ml.

### Identification of circulating immune cells

Mice were inoculated with 1.25 × 10^5^ B16 cells and treated with 200 μg of BLS or PBS 2 days later. Blood was collected via the submandibular vein into tubes with heparin (10% of final volume), 3 h after BLS treatment. Red blood cells were lysed using RBC lysis buffer (Biolegend) and single cell suspensions were used for flow cytometry staining.

### Identification of infiltrating lymphocytes

Mice receiving 1.25 × 10^5^ B16 cells were treated with 200 μg of BLS at day 2 or 10 post tumor inoculation. At day 14 post tumor inoculation, mice were sacrificed and tumors resected. Excised tumors were disrupted and passed through a 30-mm pore filter (Pre-separation filters, Miltenyi Biotec) to obtain a single-cell suspension.

### Flow cytometry

Staining antibodies are detailed in Table S1. To assess viability, cells were stained with Fixable Viability Stain 660 (BD Biosciences, San José, CA, USA). Cells were then stained in 10% FCS for 30 min at 4 °C, and fixed in 4% paraformaldehyde (PFA). For regulatory T cell detection, cells were fixed and permeabilized using the FoxP3 staining kit (eBioscience, San Diego, California, United States), following manufacturer’s instructions. Cells were acquired on a FACSCalibur cytometer (BD Biosciences, San José, CA, USA) and data were analyzed by using CellQuest software (BD Immunocytometry Systems, San José, CA, USA).

### Generation of mouse BMDC

BMDC were generated according to the method of Lutz et al.^48^. Briefly, femurs and tibiae of C57BL/6J mice were removed and freed of muscles and tendons. The bones were placed in 70% ethanol for 2 min and subsequently washed in PBS. Both bone ends were cut off, and the marrow was flushed out with RPMI 1640 medium. The cells were centrifuged for 8 min at 360*g*. The cells were seeded in bacterial petri dishes at a density of 2 × 10^5^/ml in 10 ml of RPMI 1640 with 2 nM l-glutamine, 100 U of penicillin/ml, 100 µg of streptomycin, 50 µM 2-ME, and 10% FBS supplemented (R10 medium) with 5% mouse GM-CSF (mGM-CSF)-containing supernatant from a J558 cell line stably transfected with mGM-CSF. On day 3 of culture, another 10 ml of R10 medium with mGM-CSF was added. On days 6 and 8, the culture supernatant was collected and centrifuged, and the cell pellet was resuspended in 20 ml of R10 medium with mGM-CSF. On day 9, nonadherent cells were collected by gentle pipetting, centrifuged at 300*g* for 10 min, and resuspended in R10 medium; 85% of these cells were CD11c positive (data not shown). For stimulation, BMDC were cultured for 18 h in R10 medium with 90 µg of BLS, preincubated with PMB-agarose.

### Tissue processing and slicing

Tumors were fixed in 4% paraformaldehyde (Electron Microscopy Sciences) for 2 h at room temperature. Following fixation tumors were removed en bloc and collected into HBSS containing 25 mM HEPES. A Compresstome VF-300 vibratome (Precisionary Instruments, Greenville, USA) was used to cut 200 µm slices in the horizontal plane of the tumor. Slices were collected into PBS-BSA-azide. Primary staining with antibodies against CD45 (PE-Dazzle; 30-F11) purchased from Biolegend and phalloidin-Alexa 488 (Thermo Fisher Scientific). Staining was performed in the presence of purified anti-mouse CD16/CD32 (Fc block) for overnight in a humidified incubator at 4 °C. Prior to image acquisition tumor slices were stained with DAPI (Sigma-Aldrich) at 1:2000 in PBS for 10 min at room temperature and protected from light.

### Microscopy of precision-cut tumor slices: image acquisition and analysis

Slices were imaged in 24-well µ-plates (ibidi, Martinsried, Germany) using a Leica SP5 MP/FILM inverted microscope (Leica, Wetzlar, Germany) with a 20× objective. Z stacks composed of ten individual images and covering approx. 50–100 µm thickness were acquired with a 1024 × 1024 µm field of view, and z-stack projection and background correction of images were performed in Imaris software Bitplane 8.1.

### Ethics approval

All procedures in this study were done at Instituto Leloir, Buenos Aires, Argentina, and were carried out in strict accordance with the recommendations from the Guide for the Care and Use of Laboratory Animals of the National Institutes of Health. All protocols were approved by the Committee on the Ethics of Animal Experiments of the Leloir Institute (Protocol #FG58/2011) and were carried out in compliance with the ARRIVE guidelines. All efforts were made to minimize suffering. Mice were monitored at least every 2 or 3 days and sacrificed by cervical dislocation when tumors reached a volume greater than 3000 mm^3^, when tumors where ulcerated or signs of discomfort were observed. A minimum of six and a maximum of ten mice were used per group for each experiment; the total number of mice used for this work was approximately 136.

### Statistical analysis

GraphPad PRISM 5.0 software (GraphPad) was used for statistical analyses. All the experiments were carried out in duplicate or triplicate and data was pooled. Statistical significance was set at (*p < 0.05, **p < 0.01 and ***p < 0.001). To determine statistical significance between samples, *t* tests were performed. Survival curves were estimated using a Kaplan–Meier plot and compared using the log-rank test. FACS results are expressed as means ± SEM; tumor volumes are shown as means ± SEM. Levels of significance were determined using unpaired two-tailed Student's t-test.

## Supplementary Information

Supplementary Information.

## Abbreviations

BLS: Brucella lumazine synthase
TLR: Toll-like receptor
PD-1: Programmed cell death protein-1
PD-L1: Programmed cell death ligand-1
Treg: Regulatory T cells
MDSC: Myeloid-derived suppressor cells
DC: Dendritic cells
BMDC: Bone-marrow dendritic cells
CTL: Cytotoxic T lymphocytes
TME: Tumor microenvironment
ICB: Immune checkpoint blockade
ICOS: Inducible T-cell costimulator
FOV: Field of view
CTLA-4: Cytotoxic T-lymphocyte-associated protein-4
TIM-3: T-cell immunoglobulin mucin-3
