# Supplementary material for: A TLR4 agonist improves immune checkpoint blockade treatment by increasing the ratio of effector to regulatory cells within the tumor microenvironment
Source: Sci Rep. 2021 Jul 28;11:15406. doi: 10.1038/s41598-021-94837-7 (PMC8319313; doi:10.1038/s41598-021-94837-7)
Supplement: Supplementary file 1 — Supplementary Information. [file 41598_2021_94837_MOESM1_ESM.docx]

**A TLR4 agonist improves immune checkpoint blockade treatment by increasing the ratio of effector to regulatory cells within the tumor microenvironment**

*Farias A^1^, Soto A^1^, Puttur F^2^, Goldin CJ^1^, Sosa S^1^, Gil C^1^, Goldbaum FA^1^, Berguer, PM^1*^*

^1^ Fundación Instituto Leloir, IIBBA, Consejo Nacional de Investigaciones Científicas y Técnicas (CONICET), Buenos Aires, Argentina

^2^ Inflammation, Repair and Development, National Heart and Lung Institute, Imperial College London, London, UK.

*^*^*Corresponding author: [pberguer@leloir.org.ar](mailto:pberguer@leloir.org.ar)

**Additional Information**


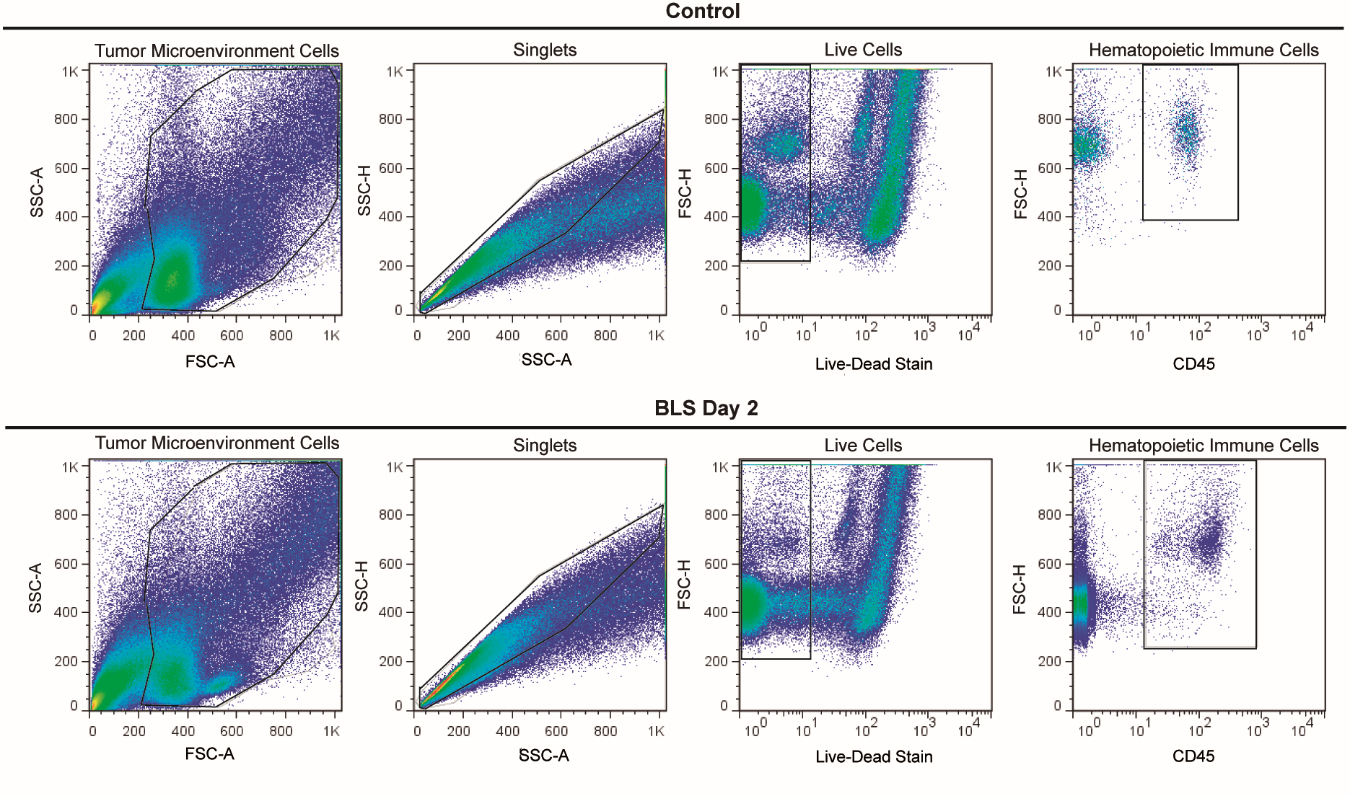


**Fig. S1. Gating strategy for identification of hematopoietic immune cells within the tumor, related to Fig. 1.** C57BL/6J mice were inoculated with 1.25x10^5^ B16 cells subcutaneously in the right flank. At day 2, 200 μg of BLS or PBS (control) were subcutaneously administered in the base of the tail. At day 14 the abundance of CD45^+^ cells within the tumor was analyzed by flow cytometry. TIL were determined as CD45^+^ cells, pre-gated based on the SSC-A vs FSC-A dot plot and live cells (determined using a fixable live dead dye).


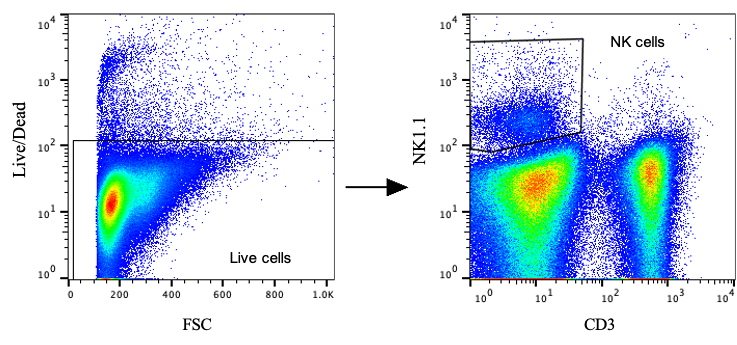


**Fig. S2. Gating strategy for identification of hematopoietic immune cells present in the blood, related to Fig. 3.** C57BL/6J mice were inoculated with 1.25x10^5^ B16 cells subcutaneously in the right flank. At day 2, 200 μg of BLS or PBS (control) were subcutaneously administered in the base of the tail and 3h later, blood was obtained, and abundance of NK cells was analyzed by flow cytometry.


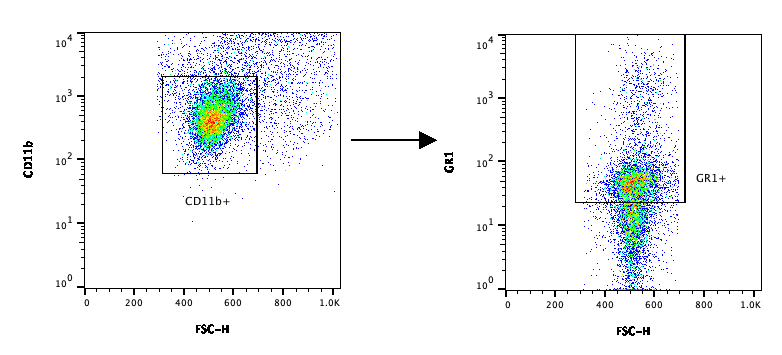


CD11b

FSC-H

Gr1

FSC-H

MDSC cells


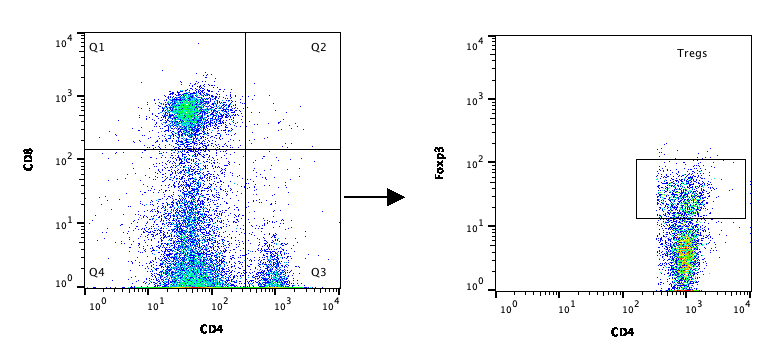


CD8

CD4

FoxP3

CD4

Tregs

CD8^+^ T cells

CD4^+^ T cells

(A)

(B)

**Fig. S3. Gating strategy for identification of hematopoietic immune cells within the tumor, related to Fig. 4.** C57BL/6J mice were inoculated with 1.25x10^5^ B16 cells subcutaneously in the right flank. At day 2, 200 μg of BLS or PBS (control) were subcutaneously administered in the base of the tail. At day 14 the abundance of CD8^+^ and CD4^+^ T cells as well as Treg were analyzed from CD45^+^ CD3^+^ cells (A). The proportion of MDSC cells from CD45^+^ CD11b^+^ cells (B) within the tumor was analyzed by flow cytometry.


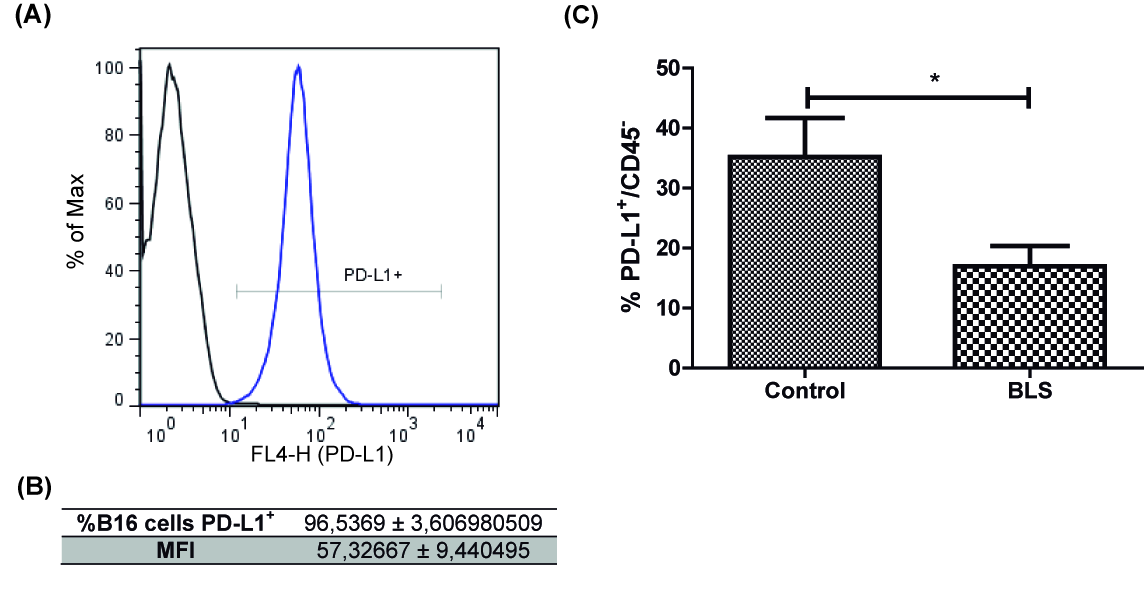


**Fig S4. Expression of PD-L1 in the non-immune cells within the tumor diminishes upon administration of BLS.** Expression of PD-L1 on B16 cells *in vitro* was assessed by flow cytometry. (A) A representative histogram is shown. (B) Percentage of PD-L1^+^ cells and level of expression of PD-L1 was quantified from three independent experiments (+ SD). (C) To study expression of PD-L1 in the TME and the effect of treatment with BLS at day 2, PD-L1 expression in CD45^-^ cells was assessed at day 14 by flow cytometry. Data from two independent experiments have been pooled (3 replicates per experiment), *p<0.05.

**Table S1. Antibodies used for flow cytometry.**

| **Antibody** | **Clone** | **Fluorochrome** | **Target species** | **Manufacturer** |
| --- | --- | --- | --- | --- |
| CD45 | 30-F11 | PerCP Cy5 | Mouse | BD Biosciences |
| CD3e | 145-2c11 | APC-Cy7 | Mouse | eBioscience |
| CD8 | 53-6.7 | FITC | Mouse | BD Biosciences |
| CD4 | H129.19 | APC | Mouse | BD Biosciences |
| Foxp3 | MF23 | Alexa Fluor 488 | Mouse | BD Biosciences |
| ICOS | 7E.17G9 | PE | Mouse | eBioscience |
| CD11b | M1/70 | APC | Mouse | eBioscience |
| Gr1 | RB6-8C5 | PE | Mouse | eBioscience |
| CD3 | 17A2 | FITC | Mouse | BioLegend |
| NK1.1 | PK136 | PE | Mouse | BD Biosciences |
| CD11c | HL3 | PE-Cy7 | Mouse | BD Biosciences |
